# Pericardial Effusion Secondary to Nilotinib in an Elderly Patient With Chronic Myelogenous Leukemia

**DOI:** 10.7759/cureus.23855

**Published:** 2022-04-05

**Authors:** Geetika Arora, Paulus Adinugraha, Amna Aijaz, Alvaro Vargas Pelaez, Maurice Rachko

**Affiliations:** 1 Internal Medicine, Mount Sinai Beth Israel/Icahn School of Medicine at Mount Sinai, New York City, USA; 2 Internal Medicine/Public Health, George Washington University, Washington, D.C., USA; 3 Cardiology, Mount Sinai Beth Israel/Icahn School of Medicine at Mount Sinai, New York City, USA

**Keywords:** cardiac tamponade, serositis, pericardial effusion, tyrosine kinase inhibitors (tki), nilotinib

## Abstract

Tyrosine kinase inhibitors (TKIs) are the first-line treatment for patients with chronic myelogenous leukemia (CML). Serositis, including pleural and pericardial effusions, is a frequent adverse event with some TKIs while less frequent with others. We present a case of a 76-year-old woman with CML on nilotinib who presented with progressive fatigue and was eventually found to have cardiac tamponade from a large pericardial effusion attributed to nilotinib. The patient required urgent therapeutic pericardiocentesis and switching of TKIs from nilotinib to bosutinib.

## Introduction

Chronic myelogenous leukemia (CML) is a clonal myeloproliferative disease characterized by a cytogenetic abnormality called the Philadelphia (Ph) chromosome. The Ph chromosome is formed by a reciprocal translocation involving chromosomes 9 and 22 and results in an oncogenic fusion gene encoding BCR-ABL. The BCR/ABL tyrosine kinase inhibitor (TKI) is an established standard of therapy in CML and long-term therapy with TKI has resulted in improved outcomes for these patients [1]. While older generation TKIs (e.g., imatinib) have been associated with adverse effects such as pleural effusions, the effect of newer generation TKIs (e.g., nilotinib) on the development of serositis is less known. We present a case report of a patient who developed pleural and pericardial effusion after treatment with nilotinib.

## Case presentation

**Figure 1:**
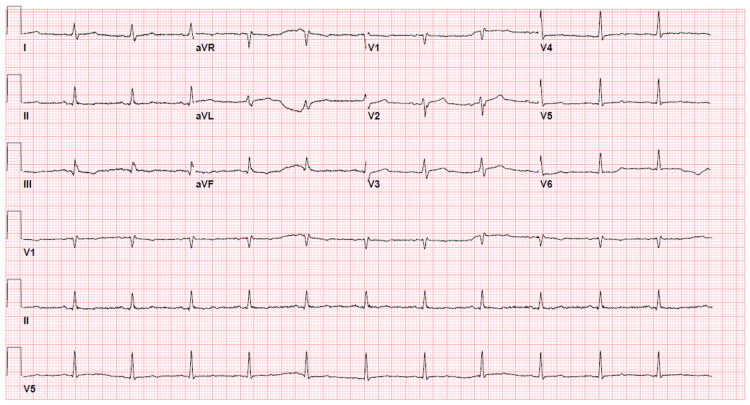
EKG at the time of presentation depicting low voltage QRS complexes.

**Figure 2:**
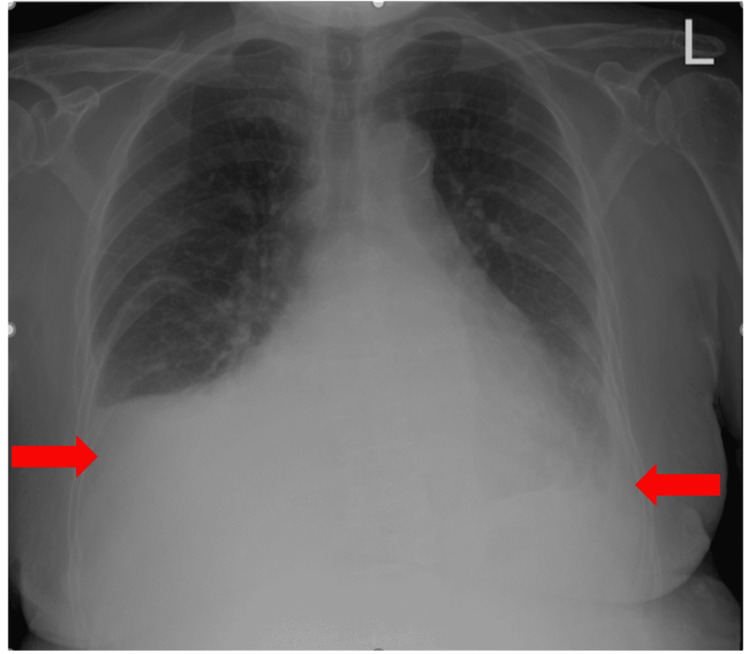
Chest x-ray depicting large right and small left pleural effusion.

**Figure 3:**
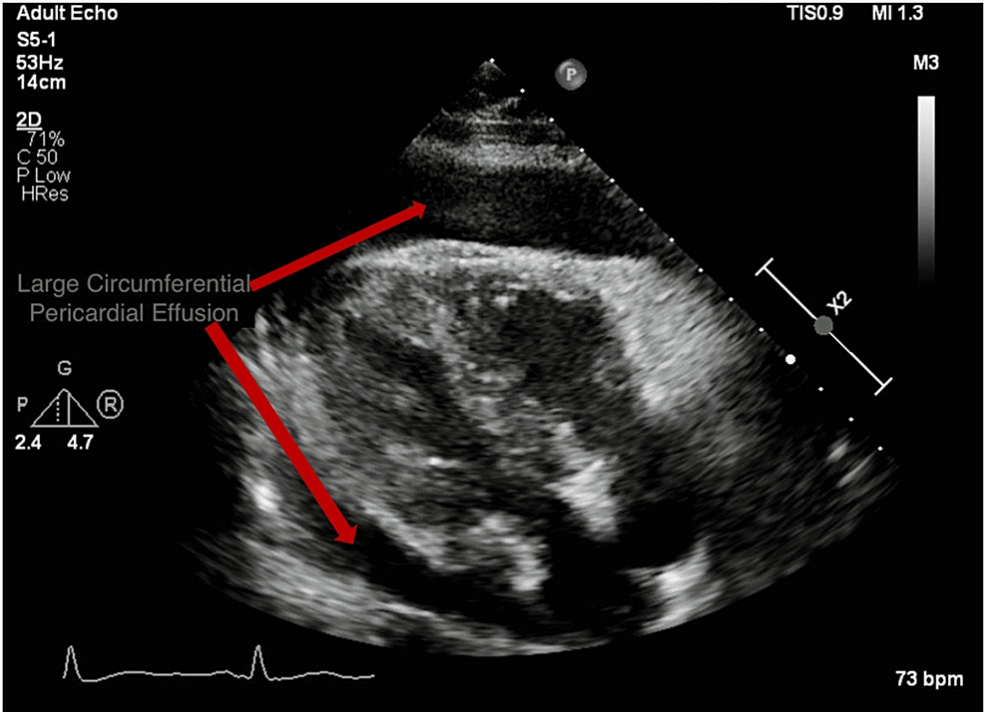
Transthoracic echocardiogram depicting large pericardial effusion.

**Figure 4:**
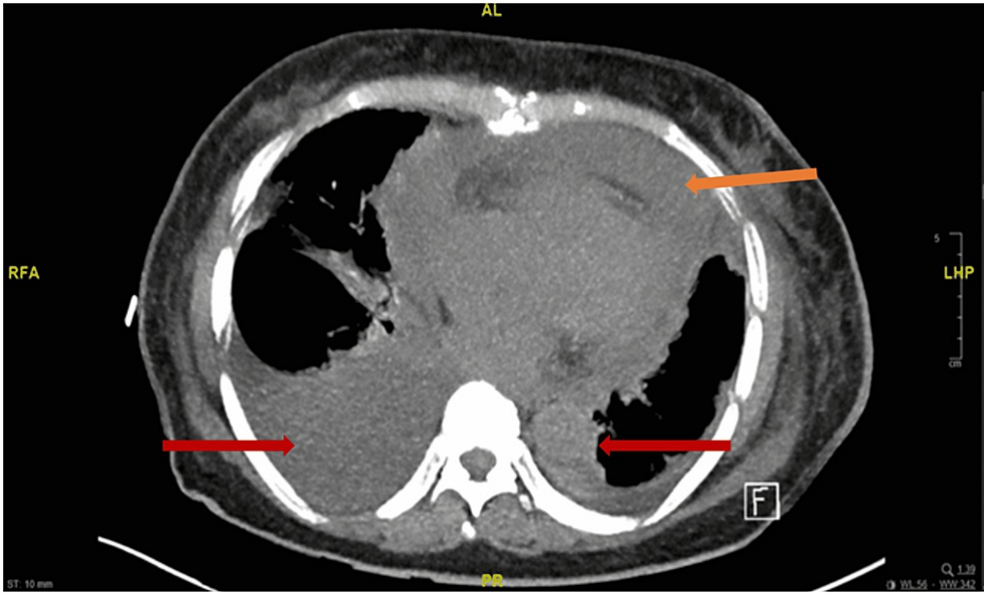
CT scan of the chest confirming the large pericardial effusion (orange arrow) and a large right and small left pleural effusion (red arrow).

A 76-year-old woman with a known three-year history of CML, hyperlipidemia, and hypertension presented with progressive fatigue for one week and anuria for one day. One year ago, after failing treatment with imatinib and dasatinib due to adverse effects (pleural effusions), she was switched to nilotinib. Over the course of one year, she tolerated the medication without any adverse effects. On presentation, her blood pressure was 96/56mm Hg with a regular heart rate of 74 beats per minute, respiratory rate of 16 per minute, and oxygen saturation around 100% on room air. On auscultation, breath sounds were decreased on the right side of the chest and heart sounds were notably distant, with jugular venous distention up to 12cm H_2_O. Electrocardiogram (EKG) was notable for a new finding of low-voltage QRS complexes (Figure 1). An x-ray of her chest showed large right and small left pleural effusions (Figure 2). Bedside ultrasound showed a large pericardial effusion suggestive of cardiac tamponade and bilateral B-lines (Figure 3). A computerized tomography (CT) scan of the chest confirmed the large pericardial effusion and a large right-sided pleural effusion (Figure 4). Initial laboratory values showed acute kidney injury with serum creatinine of 6.42mg/dl and hyperkalemia (serum potassium of 7.5mEq/L). More comprehensive laboratory results are shown in Table 1.

**Table 1:** Laboratory values. AST: aspartate aminotransferase; ALT: alanine aminotransferase; BUN: blood urea nitrogen; pCO2: partial pressure of carbon dioxide; HCO3: bicarbonate

	One month before admission	On the day of presentation	Nine days after presentation
Creatinine (mg/dL)	0.9	6.42	0.87
BUN (mg/dL)	18	103	16
Potassium (mmol/L)	5.1	7.5	4.5
Phosphorus (mg/dL)	3.4 (three years ago)	9.9	2.6
Calcium (mg/dL)	9.5	8.3	8.3
Uric Acid (mg/dL)	3.0 (three years ago)	15.8	2.4
AST (IU/L)	28	265	46
ALT (IU/L)	28	243	64
WBC (x10^3^/μL)	7.2	16.5	7.1
Hemoglobin (g/dL)	9.6	10.6	9.6
Platelet (x10^3^/μL)	140	395	194
pH, Ven	none	7.15	7.52
pCO2, Venous (mm Hg)	none	35	33
HCO3 (mmol/L)	23.6	10	26.4
Lactate, Venous (mmol/L)	none	4.1	0.9

The patient underwent emergent pericardiocentesis with the removal of 850ml of sanguineous pericardial fluid and drain placement. An additional 800ml of serosanguineous fluid was drained over the next 48 hours. Pericardial fluid analysis revealed 1722 white blood cells (55% neutrophils, 38% lymphocytes, 6% monocytes, 1% eosinophils) and 774,000 red blood cells. The pericardial fluid culture showed no growth. Cytopathology and flow cytometry showed no evidence of a lymphoproliferative disorder or leukemia. All other infectious workup done on serum and pericardial fluid was negative. 

For her right-sided pleural effusion, thoracentesis was performed, which drained 1400ml of transudative fluid. Pleural fluid culture also showed no growth. Cytopathology and flow cytometry revealed no evidence of lymphoproliferative disorder. The pleural effusion that was drained a year prior was similar to this presentation. Given that alternative etiologies were ruled out, both her pericardial and pleural effusions were attributed to nilotinib.

The patient became hemodynamically stable with the resolution of metabolic abnormalities after drainage of her pericardial effusion. Upon discharge, given this life-threatening complication from nilotinib therapy, the decision was made to switch to a different TKI, bosutinib. Follow-up imaging six months later showed reaccumulation of a moderate unilateral pleural effusion, but no pericardial effusion was noted on transthoracic echocardiography. 

## Discussion

CML is a hematopoietic neoplasm characterized by uncontrolled myeloproliferation, basophilia, and the BCR/ABL oncoprotein. The BCR/ABL TKI is an established standard of therapy in the chronic phase of CML [1]. Currently, there are five TKIs approved by the United States Food and Drug Administration (FDA) available for CML: imatinib, dasatinib, nilotinib, bosutinib, and ponatinib [2]. 

Serositis is a rare, but important, side effect of the family of TKIs and has been previously reported in clinical trials. The incidence of serositis ranges from 10-48% with dasatinib, the agent most often associated with pleural effusions [3-5]. Other agents such as imatinib, nilotinib, and bosutinib have a lower incidence of serositis (<1%) [6,7]. Nilotinib-induced isolated pericardial effusion has been reported only once in literature [8]. To the best of our knowledge, this is the second case reporting nilotinib-induced pericardial effusion in a CML patient.

Although the mechanisms underlying the development of pleural and pericardial effusions with the use of TKIs have not been fully elucidated, several important correlations have been observed linking TKIs to serositis. One of the implicated pathogenetic pathways includes off-target tyrosine kinase inhibition. Dasatinib, the agent most often associated with effusions, is known to have multiple off-target effects including inhibition of platelet-derived growth factor receptor beta (PDGFR-β). In contrast, TKIs with a low incidence of serositis, such as imatinib, nilotinib, and bosutinib are all known to be weaker inhibitors of PDGFR-β [9]. The PDGFR-β receptor is involved in the regulation of angiogenesis and maintenance of interstitial fluid pressure, and its inhibition could potentially lead to seepage of fluid from the serosal layers.

The constitutively active BCR-ABL tyrosine kinase is the defining molecular abnormality in Ph+ CML. The pathogenic role of BCR-ABL in CML provided the rationale for therapeutic targeting of this signaling protein. Imatinib being the first available BCR-ABL targeted therapy became the standard first-line therapy for CML in the chronic phase. Recently, the involvement of Src family Kinase (SFK) in BCR-ABL signaling, the transforming activity of BCR-ABL, progression of CML to blast crisis, and Imatinib resistance has been proven, thus prompting the use of agents with dual BCR-ABL/SFK inhibition such as dasatinib providing added therapeutic advantage. However, in addition to involvement in CML progression, SFKs also regulate focal adhesions and adherens junctions, two subcellular cell-matrix attachment structures key in regulating cell adhesion that might play a role in the stability of the pleural/pericardial epithelium [10,11]. These kinases are over-expressed in the pulmonary vasculature, pleural, and/or pericardial epithelium. Additionally, the vascular permeability activity mediated by vascular endothelial growth factor (VEGF) is directly dependent on the SFKs, which are widely expressed in lung tissue and endothelial surfaces [12]. Inhibition of SFKs could lead to alteration in vascular permeability and intra-cellular junction related epithelial stability leading to leakage of serosal fluid,

Another possible pathway linking TKIs to serositis is an immune-mediated mechanism responsible for the development of serosal inflammation [13]. TEC kinase and Bruton's agammaglobulinemia tyrosine kinase (BTK), both targets for TKIs, are involved in the signaling pathway of the T- and B-cell receptors as well as in endothelial, pulmonary, and mast cells [14]. The immuno-pathogenetic mechanism for pleural/pericardial effusions with TKI use is supported by the association of effusions in patients with a history of autoimmune disease and the historical response of highly symptomatic patients to steroids [13]. Risk factors for the development of TKI-related serosal inflammation include a history of previous cardiac disease, hypertension, hyperlipidemia, autoimmune disease, or a previous history of rash in response to TKI therapy [15]. Previous studies have also shown that the timing of the development of serosal inflammation can vary from three weeks after starting therapy to up to two years [15]. While the most common serosal inflammation noticed is pleural effusion, concurrent pericardial effusion is noted in up to 29% of patients treated with dasatinib [13].

In our case, the patient developed both pleural and pericardial effusions while being on treatment with nilotinib. Of note, nilotinib does not inhibit SFK, but it does inhibit PDGFR-beta and thus that along with the immunologic pathway could explain the pericardial effusion in our patient. 

Identification of the etiology and pericardiocentesis is imperative in patients with a history of malignancy to rule out both infection and recurrent malignancy. In cases where a negative workup is obtained, withdrawal of the TKI and/or changing to a different TKI within the same class prevents a recurrence of serosal inflammation. Our patient was switched to bosutinib and follow-up showed recurrence of her pleural but not pericardial effusion.

## Conclusions

Pericardial effusion is a rare but life-threatening complication that must be considered in any patient on BCR-ABL inhibitors, even with a less likely agent such as nilotinib in our case. In addition to management of the acute pericardial effusion, removal of the offending TKI agent and switching to another newer generation BCR-ABL inhibitor with less off-target inhibition should be considered. CML patients on BCR-ABL inhibitor therapy require long-term surveillance, and physicians need to be cognizant of this potentially life-threatening complication. Further studies are warranted to evaluate the cost-effectiveness of screening echocardiograms in this population.
